# Metal-Ion-Doped Manganese Halide Hybrids with Tunable Emission for Advanced Anti-Counterfeiting

**DOI:** 10.3390/nano13121890

**Published:** 2023-06-20

**Authors:** Chun Sun, Hu Zhang, Zhihui Deng, Chao Fan, Xiaohui Liu, Mingming Luo, Yiwei Zhao, Kai Lian

**Affiliations:** 1 Tianjin Key Laboratory of Electronic Materials and Devices, School of Electronics and Information Engineeing, Hebei University of Technology, 5340 Xiping Road, Tianjin 300401, Chinalk981231@126.com (K.L.); 2 Baotou Teachers’ College, Inner Mongolia University of Science and Technology, Baotou 014020, China; 3 Zhejiang Ruico Advanced Material Co., Ltd., No. 188 Liangshan Road, Huzhou 313018, China

**Keywords:** anti-counterfeiting, doping, manganese halides, stimuli-responsive, photoluminescence

## Abstract

Stimuli-responsive luminescent materials have received great attention for their potential application in anti-counterfeiting and information encryption. Manganese halide hybrids have been considered an efficient stimuli-responsive luminescent material due to their low price and adjustable photoluminescence (PL). However, the photoluminescence quantum yield (PLQY) of PEA_2_MnBr_4_ is relatively low. Herein, Zn^2+^- and Pb^2+^-doped PEA_2_MnBr_4_ samples are synthesized, and show an intense green emission and orange emission, respectively. After doping with Zn^2+^, the PLQY of PEA_2_MnBr_4_ is elevated from 9% to 40%. We have found that green emitting Zn^2+^-doped PEA_2_MnBr_4_ could transform to a pink color after being exposed to air for several seconds and the reversible transformation from pink to green was achieved by using heating treatment. Benefiting from this property, an anti-counterfeiting label is fabricated, which exhibits excellent “pink-green-pink” cycle capability. Pb^2+^-doped PEA_2_Mn_0.88_Zn_0.12_Br_4_ is acquired by cation exchange reaction, which shows intense orange emission with a high QY of 85%. The PL of Pb^2+^-doped PEA_2_Mn_0.88_Zn_0.12_Br_4_ decreases with increasing temperature. Hence, the encrypted multilayer composite film is fabricated relying on the different thermal responses of Zn^2+^- and Pb^2+^-doped PEA_2_MnBr_4_, whereby the encrypted information can be read out by thermal treatment.

## 1. Introduction

Counterfeiting and forgery are an ever-growing global issue, causing serious financial losses to governments, companies and individuals. Over the last few decades, counterfeit products have been widely found in daily consumer goods, diplomas, medicines and banknotes [1,2,3]. Information security, including encryption and anticounterfeiting, is becoming an important task that needs to be urgently accomplished. The development of advanced anti-counterfeiting technologies is the only way to solve this dilemma. Due to the merit of visual identifiability and easy operation, luminescent anti-counterfeiting is considered to provide ideal security elements [4,5].

However, the current luminescent materials are easy to mimick due to their monotony response to the excitation light. Stimuli-responsive luminescent materials and multi-mode excited luminescent materials as new kinds of advanced anti-counterfeiting luminescent materials have attracted much attention. Multi-mode excited luminescent materials show multicolor emissions under multiple excitation modes (PL, upconversion luminescence, and long-lasting luminescence), which is hard to mimick [6,7]. However, multi-mode excited luminescent materials usually contain expensive rare earth elements and require different excitation light sources, which is severely restricted by non-portable testing tools. Stimuli-responsive luminescent materials will change their optical properties, including spectra and lifetime, in response to external stimuli such as light, [8] electricity [9], temperature [10,11], force [12], and gases [13]. So far, various organic and inorganic luminescent materials, including organic dyes [14], quantum dots [15,16,17], and transition metal complexes [9], have been used as stimuli-responsive PL materials, but most of these materials suffer from complicated and tedious synthesis and unsatisfactory PL properties.

The newly emerging luminescent materials lead halide perovskites have received great attention due to their fascinating properties such as low cost, high PLQY, and adjustable emission spectrum [18,19,20,21,22,23,24,25,26,27]. Benefiting from their intrinsic vulnerability toward the external environment, some studies have also shown that perovskites could be exploited in anti-counterfeiting, confidential information encryption, and decryption [15,16,17,28]. However, the toxicity of lead and low crystal stability severely impede their further application in information security. To address these issues, much effort has been devoted to developing lead-free perovskites by replacing Pb^2+^ with Sn^2+^, Ge^2+^, Bi^2+^, and so on. Although Sn^2+^ and Ge^2+^ ions have similar electronic structures to Pb^2+^, tin-based and germanium-based perovskites possess a relatively low PLQY and they are easily oxidized in the air [29,30]. Other lead-free perovskites including Sb-based Cs_4_CuSb_2_Cl_12_ [31], bi-based Cs_3_Bi_2_X_9_ [32], and double perovskites Cs_2_AgBiBr_6_ [33], and Cs_2_AgInCl_6_ [34], have low defect tolerance and show unsatisfactory PL performance.

Due to the low toxicity, low price, and high abundance of Mn^2+^, manganese halide hybrids have been considered as an efficient luminescent material [35,36,37]. Besides, Mn^2+^ is stable in an ambient environment. The emission spectrum of manganese halide hybrids can be tuned by the crystalline field of Mn^2+^ ions. The tetrahedral coordination Mn^2+^ emits a green color with a narrow full width at half-maximum (FWHM), whereas the octahedral coordination Mn^2+^ exhibits orange emission with a broad FWHM [35,36,37]. For Mn-based material, its emission can be adjusted by the ratio of reactants due to this unique luminescent property, which is conducive to a color-changing application. Zang et al. synthesized green emitting 0D organic metal halide C_6_N_2_H_16_MnBr_4_, which could be transformed into the non-emissive hydrated phase C_6_N_2_H_16_MnBr_4_(H_2_O)_2_ by uptake of water molecules [3]. The reversible color changing was due to the change in the coordination environment. Rewritable PL paper has been constructed relying on this reversible structure transformation, which showed excellent cycle capability. Chen and co-workers found that tetrahedron and trigonal bipyramid could be converted to each other as well. Specifically, green emitting [MnBr_2_(dppeO_2_)]_n_ could transform to [MnBr_2_(dppeO_2_)(DMF)]_n_ upon exposure to DMF vapor, and reversible conversion could be realized by heating treatment [38]. Han et al. reported a tunable pure-color red/green/blue emission in cesium manganese bromides nanocrystals (NCs) by modulating their crystal field strengths. Red-emitting CsMnBr_3_ NCs could transform into green-emitting Cs_3_MnBr_5_ NCs by adding isopropanol. Furthermore, after contact with water, either CsMnBr_3_ NCs or Cs_3_MnBr_5_ NCs could transform into blue-emitting Cs_2_MnBr_4_·2H_2_O NCs, while Cs_2_MnBr_4_·2H_2_O NCs could transform into the mixture of CsMnBr_3_ and Cs_3_MnBr_5_ phase during dehydration treatment. [39]. Compared to other Mn-based halide materials, PEA_2_MnBr_4_ can be regarded as a good candidate for the stimuli-responsive luminescent materials, which does not involve complicated ligands and shows complete reversal transformation. Tang and co-workers prepared a new organic-inorganic hybrid PEA_2_MnBr_4_ single-crystal, and they found that PEA_2_MnBr_4_ possessed humidity chromism characteristics (it emitted green and pink emission at the water-desorption state and water-adsorption state, respectively) [40]. Due to the visible chromism, this PEA_2_MnBr_4_ was used as a marker to check water content (0.02 and 0.05 vol%) in toluene. However, the PLQY of PEA_2_MnBr_4_ was relatively low and the cycle stability was unsatisfactory.

Ion doping has been regarded as an effective method to optimize the properties of Mn-based materials [36,37,41]. Here, we report on the synthesis of Zn^2+^- and Pb^2+^-doped PEA_2_MnBr_4_ by the direct hot-injection method and cation-exchange method, respectively. Zn^2+^-doped PEA_2_MnBr_4_ exhibits a strong green PL band centered at 528 nm with a PLQY of 40%, and it can transform to a pink color after being exposed to air for several seconds. The reversible color change from pink to green can be realized after the heating treatment. Benefiting from this property, the anti-counterfeiting label and trademark were fabricated, which exhibited excellent “pink-green-pink” cycle capability. The Pb^2+^-doped PEA_2_Mn_0.88_Zn_0.12_Br_4_ shows broad-band orange emission originating from the ^4^T_1_–^6^A_1_ transition of octahedrally coordinated Mn^2+^ ions with a high QY of 85%. It’s worth noting that different from the Zn^2+^-doped PEA_2_MnBr_4_, the Pb^2+^ counterpart is stable in air and its PL decreases with increasing temperature. Utilizing the different thermal-responsive properties of Zn^2+^- and Pb^2+^-doped PEA_2_MnBr_4_, we have fabricated the encrypted multilayer composite film containing both Zn^2+^- and Pb^2+^-doped PEA_2_MnBr_4_. The encrypted information was concealed by the brighter orange fluorescence of Pb^2+^-doped PEA_2_Mn_0.88_Zn_0.12_Br_4_ at the upper layer and it could be read out by thermal treatment.

## 2. Materials and Methods

Materials: Zinc acetate dihydrate (ZnAc_2_, 99.9%), lead acetate (PbAc_2_, 99.99%), manganese acetate (MnAc_2_, 98%), β-phenylethylamine (PEA, 98%), tetra octyl ammonium bromide (TOAB, 98%), dodecylbenzene sulfonic acid (DBSA, 90%), and octanoic acid (OTAc, 99%) were purchased from Aladdin, Shanghai, China. Lead bromide (PbBr_2_, 99.99%) was purchased from Macklin, Beijing, China. Manganese (II) bromide (MnBr_2_, 97%) was purchased from Strem, Newburyport, MA, USA. Bromotrimethylsilane (TMSBr, 97%) was purchased from J&K, Beijing, China. Oleic acid (OA, 85%) was purchased from TCI, Shanghai, China. Xylene (95%) and hexane (97%) were purchased from Kermel, Tianjin, China.

Synthesis of PEA_2_MnBr_4_: 0.2 mmoL of MnAc_2_, 80 μL PEA, 0.3 mL DBSA, 0.2 mL OTAc and 5 mL ODE was placed into a 10 mL three-necked flask. Then, the flask was subjected to vacuum-nitrogen three times and heated to 80 °C to dissolve. After that, the flask was heated to 140 °C, and 150μL TMSBr was injected into the flask. 5 s later, the solution was cooled by an ice-water bath. The crude solution was centrifugated at 5000 rpm for 3 min. After centrifugation, the precipitate was washed with xylene twice and dried under vacuum.

Synthesis of PEA_2_Mn_0.88_Zn_0.12_Br_4_: The synthetic procedure of PEA_2_Mn_0.88_Zn_0.12_Br_4_ was similar to that of pure PEA_2_MnBr_4_, except for the addition of ZnAc_2_. The new additions of MnAc_2_ and ZnAc_2_ are 0.0294 and 0.0066 g, respectively.

Synthesis of Pb^2+^-doped PEA_2_Mn_0.88_Zn_0.12_Br_4_ by hot injection method: The synthetic procedure of Pb-doped PEA_2_Mn_0.88_Zn_0.12_Br_4_ was similar to that of PEA_2_Mn_0.88_Zn_0.12_Br_4_, except the addition of PbAc_2_. The new additions of MnAc_2_, ZnAc_2_, and PbAc_2_ are 0.0277, 0.0044, and 0.0065 g, respectively.

Synthesis of Pb^2+^-doped PEA_2_Mn_0.88_Zn_0.12_Br_4_ by cation exchange method: 0.0037 g PbBr_2_ and 0.0109 g TOAB were dissolved in 0.2 mL xylene to form a lead bromide precursor. Then, the lead bromide precursor was dropped into 3 mL of PEA_2_Mn_0.88_Zn_0.12_Br_4_ solution (0.124 g PEA_2_Mn_0.88_Zn_0.12_Br_4_ in 5 mL xylene). The solution was stirred for 10 min at room temperature. Then, the solution was subjected to centrifuge at 5000 rpm for 5 min. 5 mL of Xylene was added to the precipitate, and then centrifuged at 5000 rpm for 5 min. After that, hexane was added to wash the precipitate.

Synthesis of multilayer fluorescent composite films: 5 g of EVA was dissolved in 45 g of xylene to obtain a polymer solution. 0.2 mmol PEA_2_Mn_0.88_Zn_0.12_Br_4_ was dispersed into 2 mL polymer solution. The mixture was screen-printed on the glass substrate to print the pattern. Next, the polymer solution was spun onto the glass substrate to completely cover the pattern. Then, 0.2 mmol product of Pb^2+^-doped PEA_2_Mn_0.88_Zn_0.12_Br_4_ was dispersed into 2 mL polymer solution and spun onto the glass substrate as the upper layer. The glass was placed on the hot plate to observe the encryption and decryption process.

Characterization: The PL and PLE spectra were conducted by a FLS920P spectrometer (Edinburgh Instruments, Livingston, UK). The absolute PLQYs and time-resolved PL decays of the samples were measured by a fluorescence spectrometer (FLS920P, Edinburgh Instruments, UK). The Fourier transform infrared (FTIR) spectrum was conducted on a Thermo-Nicole iS50 FTIR spectrometer with an attenuated total reflection detector (Bruker, Bremen, Germany). The inductively coupled plasma optical emission spectrometry (ICP-OES) measurements were measured by an ICP Optima 8300 (PerkinElmer, Waltham, MA, USA). X-ray photoelectron spectroscopy (XPS) was recorded on a Thermo Scientific K-Alpha spectrometer (Thermo, Waltham, MA, USA). The X-ray diffraction (XRD) measurements were measured on a Rigaku Smart Lab 9 kW (Rigaku Corporation, Tokyo, Japan). Scanning electron microscope (SEM) images were acquired using ZEISS Sigma 500 (ZEISS, Jena, Germany).

## 3. Results and Discussion

**Figure 1:**
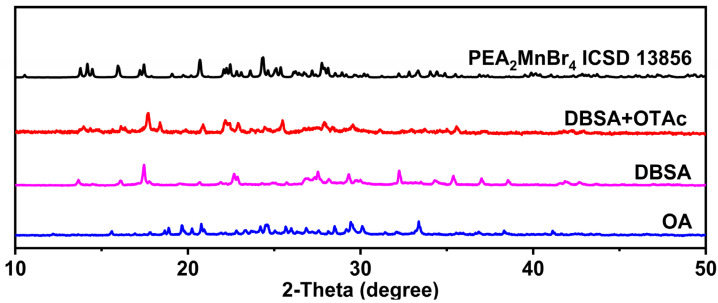
XRD patterns of PEA_2_MnBr_4_ synthesized with different ligands.

PEA_2_MnBr_4_ micro-sized powders were prepared by the hot-injection method (see details in the Materials and Methods Section). We found that the carboxylic acid ligands have an important influence on the composition of products. The X-ray diffraction (XRD) patterns of products prepared by different carboxylic acid ligands are shown in Figure 1. When oleic acid (OA) is used, the impurity phase not belonging to PEA_2_MnBr_4_ appears. Instead, most of the diffraction peaks of the product synthesized by dodecylbenzene sulfonic acid (DBSA) are consistent with the main peaks in ICSD 13,856, demonstrating that DBSA facilitates the acquisition of the PEA_2_MnBr_4_ phase. This may be because DBSA ligands have an aromatic ring structure and they can stabilize the PEA_2_MnBr_4_ phase by π-π stacking interaction with β-phenylethylamine (PEA) molecules. The strong acidity of DBSA may have a tight connection with Mn^2+^, which may further strengthen the stability of the PEA_2_MnBr_4_ phase. However, the DBSA possesses large steric hindrance, which offers incomplete protection of PEA_2_MnBr_4_, causing low PLQY and a small amount of impurity at 32.2°, 37.1°, and 38.5°. In order to elevate the PLQY and acquire the pure phase of the PEA_2_MnBr_4_, a small amount of octanoic acid (OTAc) with a short carbon-chain was added to further passivate the surface of PEA_2_MnBr_4_. As shown in Figure 1 and Figure S1, the product prepared by DBSA and OTAc possesses a pure phase structure, and the PLQY is elevated from 3% to 9%.

**Figure 2:**
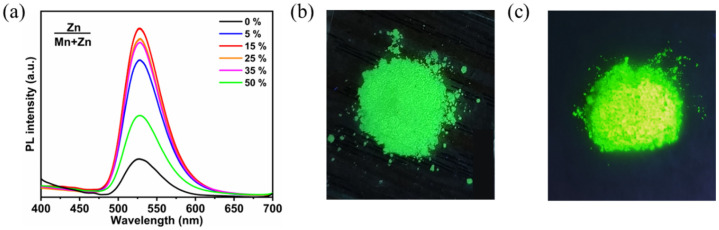
(**a**) PL spectra of PEA_2_Mn_1−x_Zn_x_Br_4_ (x = 0 − 0.5) excited at 365 nm. The photographs of PEA_2_MnBr_4_ (**b**) and PEA_2_Mn_0.88_Zn_0.12_Br_4_ (**c**) excited at 365 nm.

For further elevating the PLQY, Zn^2+^-doped PEA_2_MnBr_4_ was prepared by following the procedure of PEA_2_MnBr_4_ except adding ZnAc_2_ into the solution. Here, the feed ratios of Zn^2+^ ([Zn]/([Zn] + [Mn]) mass ratio) were 5%, 15%, 25%, 35%, and 50%. After being calibrated by inductively coupled plasma optical emission spectrometer (ICP-OES) elemental analysis, the actual ratios of Zn^2+^ were 4.6%, 12.3%, 23.6%, 32.9%, and 45.7%, respectively (Table S1). The PL spectra of PEA_2_MnBr_4_ with different Zn^2+^ ratios are shown in Figure 2a. The PL intensity of Zn^2+^-doped PEA_2_MnBr_4_ first increases with increasing Zn^2+^ concentration and reaches the maximum value at the Zn^2+^ ratio of 15% (PEA_2_Mn_0.88_Zn_0.12_Br_4_). Further increasing the substitution ratio results in a decline of PL intensity. As shown in Figure 2b,c, the PEA_2_Mn_0.88_Zn_0.12_Br_4_ shows much more intense green emission compared with the undoped PEA_2_MnBr_4_. The PEA_2_Mn_0.88_Zn_0.12_Br_4_ possesses a high PLQY of 40% compared to 9% for the pristine PEA_2_MnBr_4_. The improvement of PLQY is caused by the mitigation of concentration quenching. Because Zn^2+^ ions prefer to form a tetragonal coordination, Zn^2+^ ions can replace Mn^2+^ ions to inhibit the concentration quenching of Mn^2+^ ions, thus leading to high QY.

XRD patterns of the undoped PEA_2_MnBr_4_ and PEA_2_Mn_0.88_Zn_0.12_Br_4_ are displayed in Figure S2. All the diffraction peaks of PEA_2_Mn_0.88_Zn_0.12_Br_4_ move to a larger angle compared to that of undoped PEA_2_MnBr_4_, demonstrating that the interplanar crystal spacing is reduced. This is because the ionic radius of Mn^2+^ (0.80 Å) is slightly larger than that of Zn^2+^ (0.74 Å). Replacing Mn^2+^ with smaller Zn^2+^ leads to lattice contraction.

X-ray photoelectron spectroscopy (XPS) was carried out to analyze the electron density around the ions in PEA_2_MnBr_4_ and PEA_2_Mn_0.88_Zn_0.12_Br_4_ (Figure S3). Both the PEA_2_MnBr_4_ and PEA_2_Mn_0.88_Zn_0.12_Br_4_ show strong characteristic Mn 2p and Br 3d peaks. Only the PEA_2_Mn_0.88_Zn_0.12_Br_4_ shows the Zn 2p peak located at 1022.6 eV, proving the presence of Zn^2+^ in PEA_2_Mn_0.88_Zn_0.12_Br_4_. Besides, the binding energies of Mn 2p are reduced from 641.5 eV and 653.7 eV to 641.2 eV and 653.4 eV after Zn^2+^ doping. Introducing Zn^2+^ ions leads to the lattice contraction, which increases the electron density around the Mn^2+^ ions, thus resulting in a decrease in Mn^2+^ ion binding energy [42]. All the above characterizations suggest that Zn^2+^ ions have been successfully doped into the PEA_2_MnBr_4_.

In addition, scanning electron microscope SEM measurement was also performed to investigate the effect of Zn^2+^ doping on the morphology. As shown in Figure S4, both the PEA_2_MnBr_4_ and PEA_2_Mn_0.88_Zn_0.12_Br_4_ show irregular morphology. However, after doping with Zn^2+^, the particle size becomes smaller and the particles are more uniform than the pristine one, demonstrating that Zn^2+^ doping can retard the reaction speed and homogenize particle size distribution.

To understand the origin of emissions in PEA_2_Mn_0.88_Zn_0.12_Br_4_, excitation power dependent PL measurements were performed. As shown in Figure S5, the PL intensity is linearly increased with the excitation power density. The origin of the emission can be estimated using the power-law equation, [43]. which is defined as *I*_PL_ = *nL*^k^, where *I*_PL_ represents the PL intensity; *L* is the excitation power; coefficient *k* relates to the recombination mechanism; and *n* represents the emission efficiency. Here, the fitted *k* value is 1.1, which represents a free exciton mechanism. We have also measured the PL spectra under different excitation wavelengths. As shown in Figure S5b, all the PL spectra were fixed at the peak of 528 nm as the excitation wavelength changed from 350 to 490 nm. The independence of excitation wavelength demonstrates that the source of this green emission is attributed to the same excited state-Mn^2+^ emission center (^4^T_1_ − ^6^A_1_).

**Figure 3:**
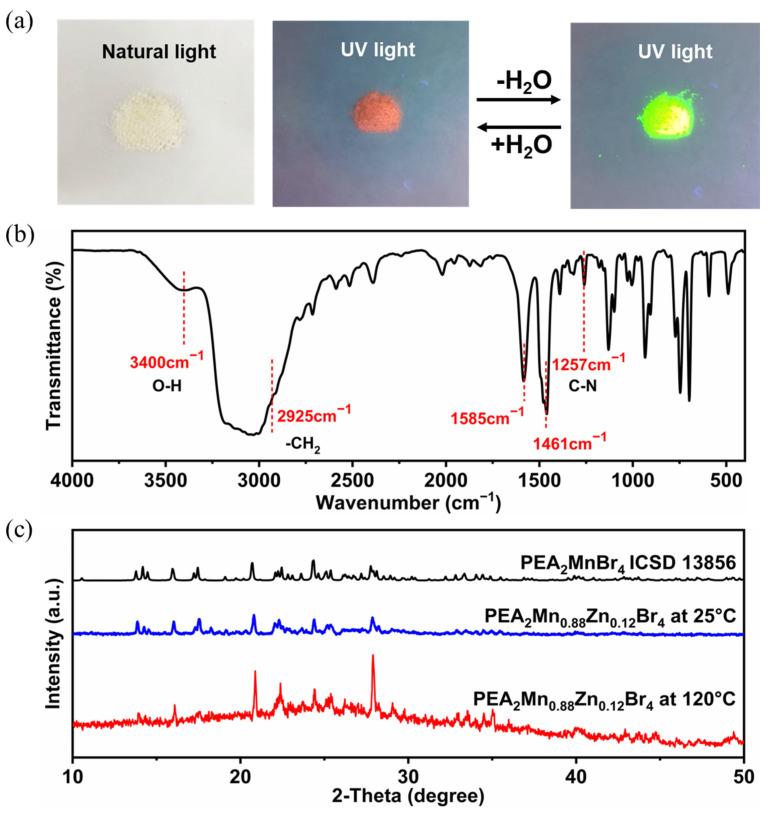
(**a**) The photographs of PEA_2_Mn_0.88_Zn_0.12_Br_4_ under natural light and UV light of 365 nm. (**b**) FTIR spectrum of PEA_2_Mn_0.88_Zn_0.12_Br_4_. (**c**) XRD patterns of PEA_2_Mn_0.88_Zn_0.12_Br_4_ at 25 °C and 120 °C.

Interestingly, green-emitting PEA_2_Mn_0.88_Zn_0.12_Br_4_ becomes pink after being placed in air for several seconds and the pink color can convert back to the green color by thermal treatment or vacuum treatment (Figure 3a). This color-changing phenomenon was associated with water adsorption and desorption, which can be confirmed by the Fourier transform infrared (FTIR) spectra results (Figure 3b). The stretching vibration peak belonging to water can be found at 3400 cm^−1^. A similar phenomenon has also been observed in PEA_2_MnBr_4_ single crystal [40]. Tang et al. believed that green and red emissions originated from tetradentate Mn^2+^ and trigonal bipyramid Mn^2+^, respectively [40]. However, detailed structure characterization was not given. Color-changing phenomenon upon contact with water was also found in C_6_N_2_H_16_MnBr_4_. Zang et al. attributed the state of water molecules adsorption to octahedron coordination geometry [3]. In order to further verify the change in the structure, we have performed XRD measurements at high temperature (120 °C) and room temperature (25 °C) (Figure 3c). Strangely, the high temperature XRD pattern of the sample was similar to the room temperature XRD pattern, except that several diffraction peaks (20.9°, 22.4° and 27.9°) in the high temperature XRD are stronger than that in room temperature XRD. Because no new diffraction peak appears, the presence of red emission cannot be accurately determined. The absence of red-emitting sample diffraction peaks may be caused by the following two reasons: first, the amount of the red-emitting sample is low; second, the crystallinity of the red-emitting sample is poor or the diffraction peaks of the red-emitting sample are much like those of the green-emitting sample. Han et al. also found that it was hard to distinguish the red-emitting CsMnBr_3_ phase by powder XRD when a small amount of red-emitting CsMnBr_3_ mixed with green-emitting Cs_3_MnBr_5_ NCs [39].

**Figure 4:**
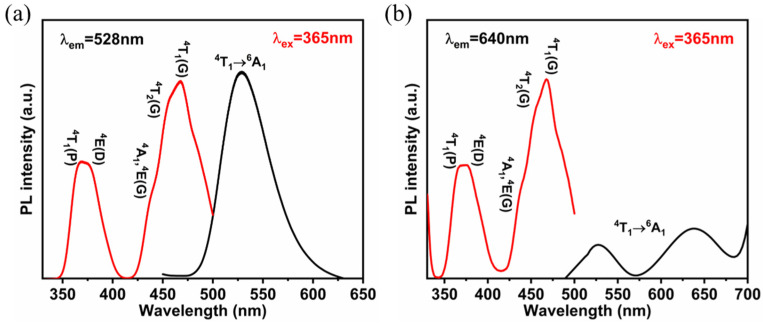
PLE and PL spectra of PEA_2_Mn_0.88_Zn_0.12_Br_4_ under water-desorption (**a**) and water-adsorption (**b**).

We can deduce that the red emission originates from octahedron coordination geometry based on the following results of Pb^2+^ doping. To get further insights into the PL mechanism, photoluminescence excitation (PLE) spectra were carried out. As shown in Figure 4, the PLE spectrum monitored at 528 nm is in coincidence with that monitored at 640 nm, demonstrating that energy transfer takes place from the tetrahedron Mn^2+^ to the octahedron Mn^2+^. A similar energy transfer process of Mn^2+^ has also been observed in [Mn(dppeO_2_)_3_] − [MnBr_4_]. [38]. The crystal field strengths around Mn^2+^ can be calculated according to the PLE spectrum. Here, the Racah parameter (B and C) and crystal field strength (Dq) of Zn^2+^-doped and undoped samples are shown in Table S2. After doping with Zn^2+^, the crystal field strength increases, which is due to the lattice contraction.

We further monitored the PL evolution of hydrated PEA_2_Mn_0.88_Zn_0.12_Br_4_ at high temperature (140 °C) and dehydrated PEA_2_Mn_0.88_Zn_0.12_Br_4_ in different humidity environments. Upon heating, the intensity of the green PL peak increases significantly and the red emission drops as time goes on (Figure S6a). After heating for 12 s, the red emission completely disappears. Further prolonging the time to 20 s, the green emission drops as well. Hence, the thermal treatment time should be 12–20 s. For the cooling process, the green-emitting PEA_2_Mn_0.88_Zn_0.12_Br_4_ was exposed to air with different humidity. The evolution of PL spectra during the cooling process was contrary to the heating process. The corresponding response time is about 100 s at a relative humidity of 38% RH. It is worth noting that the response time is related to the humidity, which is extended at relatively lower humidity. The corresponding color coordinates of color changing during the heating and cooling process are labeled and shown in Figure S6c,d.

**Figure 5:**
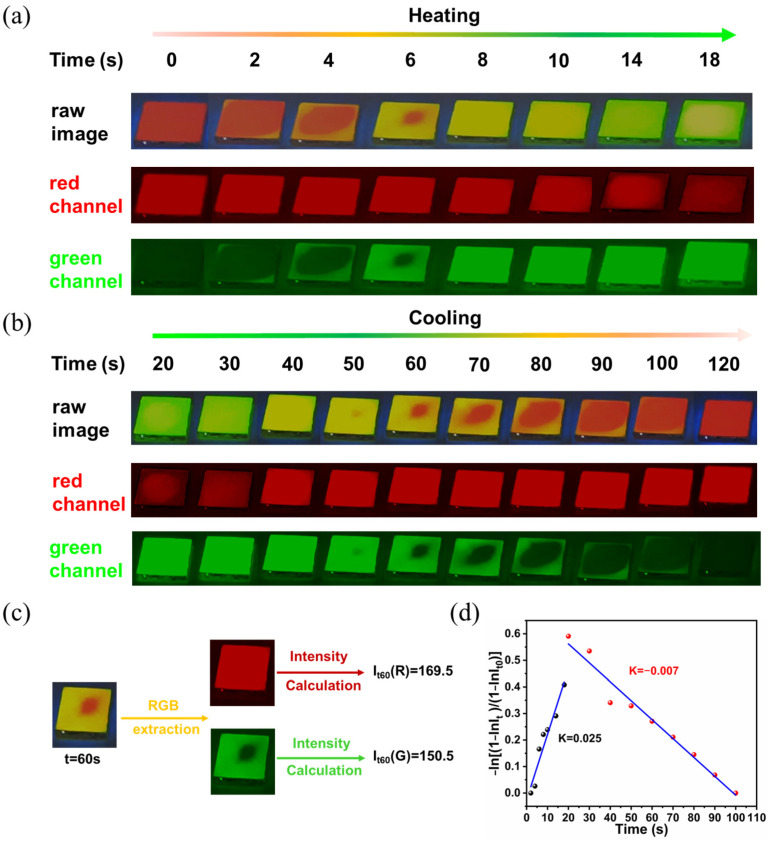
The photographs of PEA_2_Mn_0.88_Zn_0.12_Br_4_ film under UV light during the heating process (**a**) and cooling process (**b**), the corresponding red channel and green channel extracted from the photographs are shown in the middle and bottom. (**c**) The general calculation procedure of the R value (I_t_(R)) and G (I_t_(G)) value from photographs. (**d**) The calculation of the change rate of patterns during the heating process and cooling process by linear fittings.

**Figure 6:**
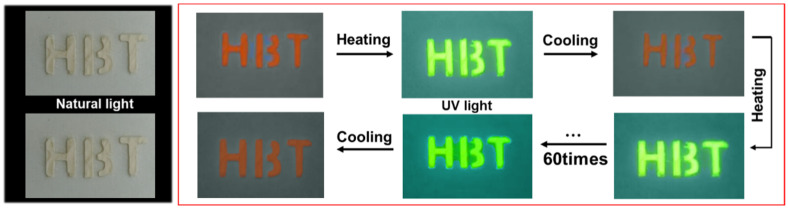
The photographs of the “HBT” letter pattern in 60 heating-cooling cycles.

The heating-cooling cycle stability was further tested. As shown in Figure S7, the emission peak, intensity, and FWHM are well maintained after 60 heating-cooling cycles, which is much better than the PEA_2_MnBr_4_ single crystal [40]. To further verify the practicability of the PEA_2_Mn_0.88_Zn_0.12_Br_4_ film, a series of images were captured at different time intervals. Here, the green channel and red channel of the images were extracted via Image J software to study the change of color (Figure 5). The color index (I_t_) can be calculated by the intensity of the red channel (I_t_(R)) and green channel (I_t_(G)) (*I_t_* = *I_t_*(G)/*I_t_*(R) + *I_t_*(G)). Also, a time-dependence curve could be drawn according to the following equation *y = −ln*[(1 − *I_t_*)/(1 − *I_t_*_0_)] = *kt*. Correspondingly, the change rates of the heating process and cooling process can be obtained, which are 0.025 min^−1^ and −0.006 min^−1^, respectively. For the anti-countering application, the letters “H”, “B”, and “T” were successfully coated on the paper, which exhibited reversible changes in green and pink emissions during the heating and cooling process. As shown in Figure 6, the “H”, “B”, and “T” still exhibit a bright green color at high temperature after 60 cycles, demonstrating excellent stability in the anti-counterfeiting application.

**Figure 7:**
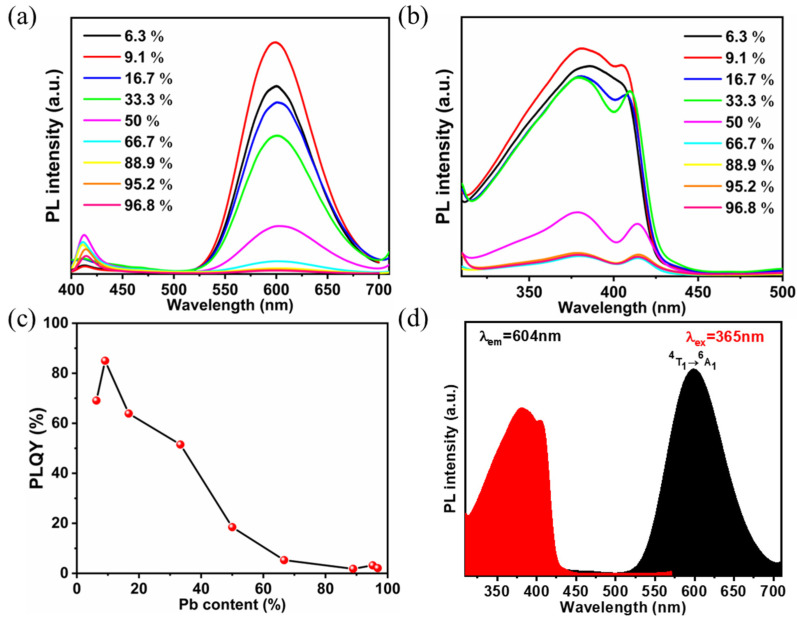
(**a**) PL spectra, (**b**) PLE spectra and (**c**) PLQY of PEA_2_Mn_0.88_Zn_0.12_Br_4_ with different Pb content. (**d**) PL and PLE spectrum of PEA_2_Mn_0.79_Zn_0.12_Pb_0.09_Br_4_.

Based on the above analysis, the variable structure of PEA_2_MnBr_4_ can be attributed to its unique 0D structure. The isolated [MnBr_4_]^2−^ tetrahedron is vulnerable to the external environment. According to the hard and soft acids and bases (HSAB) principle, the O belongs to a hard base while Mn^2+^ is a hard acid. Hence, Mn^2+^ ions prefer to bind to H_2_O to form the octahedron configuration. Because Pb-Br tends to form the octahedron structure, [PbBr_6_]^4−^ has a strong inducement effect on the transformation of [MnBr_4_]^2−^. In order to acquire highly emitting and stable octahedron PEA_2_MnBr_4_, Pb^2+^ doping was introduced. Firstly, Pb^2+^-doped PEA_2_Mn_0.88_Zn_0.12_Br_4_ was synthesized in colloidal solution by introducing PbAc_2_ into an Mn precursor. Although a bright orange emitting product can be acquired, the product is not uniform (Figure S8a). XRD measurement was performed to identify the impurity. As shown in Figure S8b, these strong diffraction peaks at 14.0°, 20.9°, and 27.9° can be indexed to PEA_2_ZnBr_4_ (CCDC 258591), suggesting that phase separation takes place due to the different activity of the Pb^2+^, Mn^2+^ and Zn^2+^ ions. In order to address the activity issue, cation exchange reaction was performed. We have tried several different combinations (PEA_2_MnBr_4_ and PEA_2_PbBr_4_, PEA_2_PbBr_4_ and MnAc_2_, PEA_2_PbBr_4_ and MnBr_2_, PEA_2_MnBr_4_ and PbBr_2_) (Figure S9) and found that the PLQYs were not high except for the combination of PEA_2_Mn_0.88_Zn_0.12_Br_4_ and PbBr_2_ (Figure S10). Hence, we have chosen the reaction of PEA_2_Mn_0.88_Zn_0.12_Br_4_ and PbBr_2_ to prepare PEA_2_Mn_0.88_Zn_0.12_Br_4_ with different Pb-doping concentrations. The actual concentrations of Pb^2+^ in the final products were determined by ICP and shown in Table S3. The PL spectra of PEA_2_Mn_0.88_Zn_0.12_Br_4_ with different Pb^2+^-doping ratios are shown in Figure 7. The highest PL intensity is acquired at the Pb^2+^ ratio of 9.1%. Actually, the PLQY of the product is as high as 85%. With the Pb^2+^ ratio changed from 6.3% to 16.7%, the lifetime becomes longer, which is consistent with the PLQY result (Figure S11).

PLE spectra were carried out to investigate the PL mechanism. As shown in Figure 7d, the energy levels of ^4^T_1_(P) and ^4^E(D) maintain at 6% substitution while energy levels of low energy excited states (^4^A_1_, ^4^E(D), ^4^T_2_(G), ^4^T_1_(G)) disappear. As the Pb^2+^ concentration changes to 9.1%, the discrete energy levels of ^4^T_1_(P) and ^4^E(D) merge together and another peak located at 414 nm appears. According to previous studies [36,44], this newly appeared peak is assigned to the lead-halide units, indicating that the electronic structure of Pb^2+^-doped PEA_2_Mn_0.88_Zn_0.12_Br_4_ is related to the interaction between [MnBr_6_]^4−^ units and [PbBr_6_]^4−^ units. Further increasing the Pb^2+^ doping concentration, the intensity of the PLE peak decreases.

**Figure 8:**
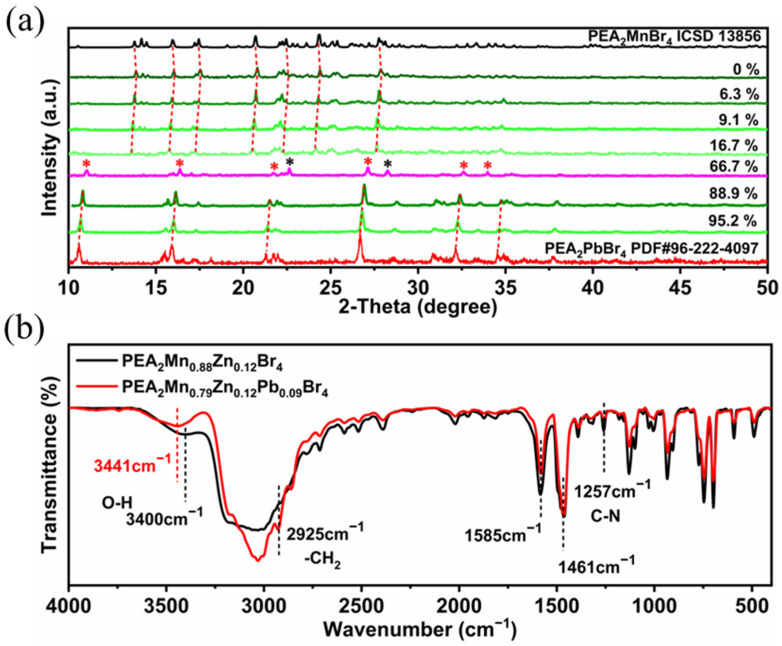
(**a**) XRD patterns of PEA_2_Mn_0.88_Zn_0.12_Br_4_ with different Pb content (Red asterisk represent the PEA_2_PbBr_4_ phase and black asterisk represent PEA_2_MnBr_4_ phase). (**b**) FTIR spectra of PEA_2_Mn_0.88_Zn_0.12_Br_4_ and PEA_2_Mn_0.79_Zn_0.12_Pb_0.09_Br_4_.

In order to investigate the effect of Pb^2+^ doping on the structure of the products, XRD was carried out (Figure 8). At low-doping concentration (6.3–16.7%), all of the peaks are still in coincidence with the patterns of PEA_2_MnBr_4_ and the diffraction peak positions shift to a smaller angle with the increasing doping concentration. Because the ionic radius of the Pb^2+^ ion is larger than that of Mn^2+^, doping Pb^2+^ ions leads to lattice expansion. As we discussed, the preservation of the PEA_2_MnBr_4_ XRD pattern indicates that the content of the octahedron Mn^2+^ is small. In addition, the intensities of several peaks (20.9°, 22.4° and 27.9°) decrease while the intensity of peaks located at 13.9°, 20.8°, and 27.8° is nearly unchanged as the doping concentration increases. Combined with the above high-temperature XRD analysis, these unchanged peaks may belong to the octahedron Mn^2+^ and these decreased peaks come from tetrahedron Mn^2+^. Further increasing to 66.7% substitution, PEA_2_PbBr_4_ appears and pure PEA_2_PbBr_4_ is acquired at 88.9% substitution. For PEA_2_PbBr_4_, the highest PLQY (3.2%) is obtained at 95.2% substitution (Figure 7a), which is much less than the Pb^2+^-doped PEA_2_Mn_0.88_Zn_0.12_Br_4_. FTIR was performed to study the influence of the Pb^2+^ doping effect. As shown in Figure 8b, the peak belonging to the stretching vibration of H_2_O shifts from 3400 cm^−1^ to 3450 cm^−1^ after doping with Pb^2+^, demonstrating that its coordination environment changes. When PEA_2_Mn_0.88_Zn_0.12_Br_4_ is exposed to air, H_2_O molecules can coordinate with Mn^2+^, changing the configuration of Mn^2+^ from tetrahedron to octahedron. Due to this coordination, the electron cloud density of O reduces, leading to the decrease in the H-O bond strength. However, when Pb^2+^ is introduced into the lattice, these unstable tetrahedrons Mn^2+^ will transform to octahedrons Mn^2+^ due to the inducive effect. In other words, the H_2_O molecules are no longer involved in the coordination of octahedron Mn^2+^. The chemical composition and electronic states of Pb^2+^-doped PEA_2_Mn_0.88_Zn_0.12_Br_4_ were further studied by XPS. As shown in Figure S12, after doping with Pb^2+^, two Pb 4f peaks at 142.3 and 138.4 eV appear, which is attributed to Pb ^4^f_5/2_, and Pb ^4^f_7/2_. Two Mn 2p peaks at 653.4 and 641.2 eV move 0.3 eV toward lower binding energy to 653.1 and 640.9 eV, and two Zn 2p peaks at 1045.6 and 1022.6 eV both move 0.5 eV toward lower binding energy to 1045.1 and 1022.1 eV. The shift of binding energy can be attributed to the Pb^2+^ incorporation that prevents the coordination of H_2_O and Mn^2+^ and changes the coordination environment of Mn^2+^. Compared to Br^−^, the O^2−^ possesses stronger electronegativity. After doping with Pb^2+^, Mn-O bonds transform to Mn-Br octahedrons, causing the increase in the electron cloud density around Mn^2+^.

**Figure 9:**
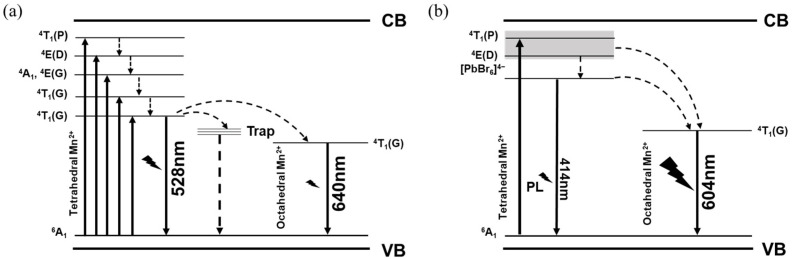
Energy states splitting and optical transitions in PEA_2_Mn_0.88_Zn_0.12_Br_4_ (**a**) and PEA_2_Mn_0.79_Zn_0.12_Pb_0.09_Br_4_ (**b**).

In order to explain the influence of the Pb^2+^ doping effect, the photophysical process of Pb^2+^-doped PEA_2_Mn_0.88_Zn_0.12_Br_4_ is depicted in Figure 9. The high PLQY of Pb^2+^-doped PEA_2_Mn_0.88_Zn_0.12_Br_4_ is mainly caused by the following three aspects: 1. energy transfer paths are optimized from tetrahedron Mn^2+^ to Pb^2+^ then to octahedron Mn^2+^. 2. the removement of H_2_O reduces the nonradiative recombination, forming perfect [MnBr_6_]^4−^. 3. Pb^2+^ doping inhibits the concentration quenching of Mn^2+^. As we discussed, the content of the [MnBr_6_]^4−^ is low in Pb^2+^-doped PEA_2_Mn_0.88_Zn_0.12_Br_4_ when the Pb^2+^ doping ratio is below 20%, but intense orange emission belonging to octahedron Mn^2+^ is observed. This can be understood by the PLE results. Both the red emission and green emission possess the same PLE spectrum, demonstrating that energy transfer occurs from tetrahedron Mn^2+^ to octahedron Mn^2+^. Interestingly, the discrete energy levels of ^4^T_1_(P) and ^4^E(D) become merged together as the Pb^2+^ concentration increases. According to the previous study [44], a quasi-continuous conduction band can be formed and spin-forbidden transition is further broken due to the merge of high energy states, which have less constraint on electron transitions, leading to increasement of the density of excitons in excited states. Besides, the transition of lead-halide units is also found in the PLE spectrum, which is placed under the energy levels of ^4^T_1_(P) and ^4^E(D). This newly appeared energy level of lead-halide precludes the energy absorption and transfer of the low energy states (^4^A_1_, ^4^E(D), ^4^T_2_(G), ^4^T_1_(G)) and directly transfers the energy to the ^4^T_1_(G). It is well known that H_2_O could easily introduce de-excitation channels for the excited state, causing severe PL decay. The removal of H_2_O can reduce the nonradiative recombination process, leading to effective radiative recombination. In addition, Pb/Mn alloying also dilutes the Mn^2+^ concentration, inhibiting the concentration quenching of Mn^2+^. All these factors optimize the energy transfer path and increase the density of excitons, thus leading to high PLQY.

**Figure 10:**
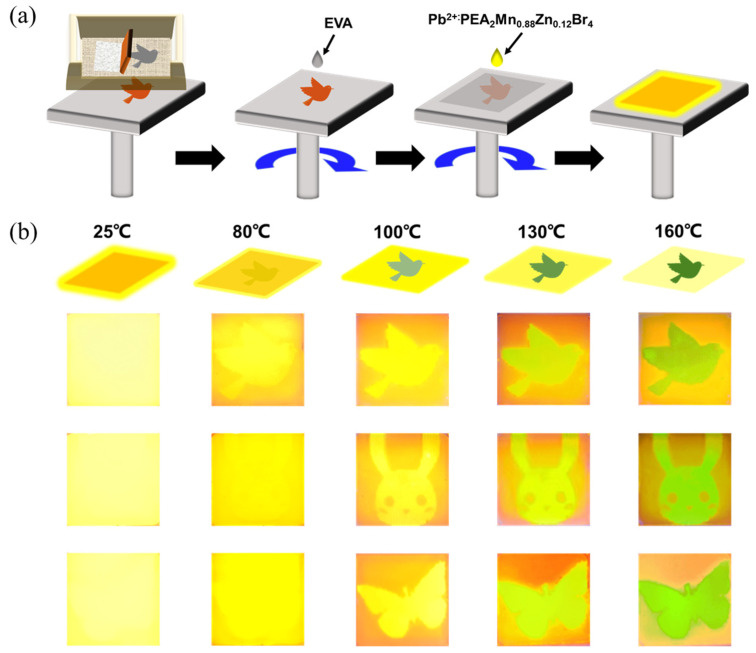
(**a**) Schematic illustration of multilayer fluorescent composite films made by screen printing technology. (**b**) The photographs of the multilayer composite film obtained at different temperatures under UV irradiation.

We have also studied the stability of the Pb^2+^-doped PEA_2_Mn_0.88_Zn_0.12_Br_4_. To our surprise, Pb^2+^-doped PEA_2_Mn_0.88_Zn_0.12_Br_4_ show excellent stability under the ambient environment, which still possesses 80% of the original PL intensity after 25 days. However, the thermal stability is totally different from the PEA_2_Mn_0.88_Zn_0.12_Br_4_. As shown in Figure S13, the PL intensity decreases sharply as the temperature increases, which is common in luminescent semiconductors. For example, the PL intensity only retains 20% of the original after heating the sample to 160 °C. The different fluorescent temperature-dependencies of PEA_2_Mn_0.88_Zn_0.12_Br_4_ and Pb^2+^-doped PEA_2_Mn_0.88_Zn_0.12_Br_4_ offer great opportunities for information encryption. Multilayer fluorescent composite films were fabricated for the application of information encryption. As shown in Figure 10, patterns of bird, rabbit, and butterfly composed of PEA_2_Mn_0.88_Zn_0.12_Br_4_ layer are screen printed on a substrate. After that, the Pb^2+^-doped PEA_2_Mn_0.88_Zn_0.12_Br_4_ solution is spin-coated on the surface of the PEA_2_Mn_0.88_Zn_0.12_Br_4_ layer to form a uniform film. The composite film exhibits strong orange emission under UV light at room temperature. When the temperature increases from 20 to 160 °C, the orange emission upper film gradually decreases and the patterns of the bird, rabbit, and butterfly with green emission appear. Finally, when the composite film is heated at 160 °C for 5 s, clear patterns of bird, rabbit, and butterfly are observed. Therefore, in this multilayer composite, the encrypted information can be read out by using a simple heating treatment. Besides, such color-changing PL behaviors can be repeated more than 10 times (Figure S14), demonstrating excellent cycle performance. This indicates that these luminescent materials can be used in document security. At room temperature the information is concealed. When we want to read the information, we can simply heat the document using a hair dryer. After the document cools to room temperature, the information can be hidden again.

## 4. Conclusions

In summary, we have fabricated Zn^2+^-doped PEA_2_MnBr_4_ and Pb^2+^-doped PEA_2_Mn_0.88_Zn_0.12_Br_4_, which possess different PL properties. For PEA_2_Mn_0.88_Zn_0.12_Br_4_, the reversible structural transformation was responsible for the color-changing phenomenon, which was due to the desorption and absorption of coordinating water. Anti-counterfeiting labels and trademarks have been constructed by taking advantage of this property, which exhibited excellent “pink-green-pink” cycle capability. Pb^2+^-doped PEA_2_Mn_0.88_Zn_0.12_Br_4_ was synthesized by the cation exchange reaction, which possessed high-efficiency orange emission. Compared to PEA_2_Mn_0.88_Zn_0.12_Br_4_, the Pb^2+^-doped PEA_2_Mn_0.88_Zn_0.12_Br_4_ was stable in the air, but its PL intensity decreased with the increase in temperature. Multilayer fluorescent composite films were fabricated by spin-coating of Pb^2+^-doped PEA_2_Mn_0.88_Zn_0.12_Br_4_ with strong orange emission onto the PEA_2_Mn_0.88_Zn_0.12_Br_4_ with weak pink emission, where the information was recorded onto the PEA_2_Mn_0.88_Zn_0.12_Br_4_ film. The encrypted information could be read out by using a simple heating treatment. In addition, the composite films showed excellent cycle stability.

## Data Availability

The data related with this work are not publicly available but can be obtained upon reasonable request from the corresponding author.

## Supplementary Materials

The following supporting information can be downloaded at: https://www.mdpi.com/article/10.3390/nano13121890/s1. Figure S1: (a)The photograph of PEA_2_MnBr_4_ synthesized with DBSA. (b) The photograph of PEA_2_MnBr_4_ synthesized with DBSA and OTAc. Table S1: Concentration ratio of Zn^2+^ in the products by ICP-OES. Figure S2: XRD patterns of PEA_2_MnBr_4_ and PEA_2_Mn_0.88_Zn_0.12_Br_4_. Figure S3: (a) XPS survey spectra of PEA_2_MnBr_4_ and PEA_2_Mn_0.88_Zn_0.12_Br_4_. High-resolutions XPS spectra of Zn2p (b) Mn2p (c) and Br3d (d) in PEA_2_Mn_0.88_Zn_0.12_Br_4_. HR-XPS spectra of Mn2p (e) and Br3d (f) in PEA_2_MnBr_4_. Figure S4: SEM images of PEA_2_MnBr_4_ (a) and PEA_2_Mn_0.88_Zn_0.12_Br_4_ (b). Figure S5: (a) Excitation power-dependent PL spectra for an excitation wavelength of 365 nm. (b) Integrated PL intensities for the emission centered at 528 nm of the PEA_2_Mn_0.88_Zn_0.12_Br_4_ in relation to the excitation power (0.08–82 μW). (c) Excitation wavelength-dependent PL spectra of PEA_2_Mn_0.88_Zn_0.12_Br_4_. Table S2: Excitation and crystal field parameters for PEA_2_MnBr_4_ and PEA_2_Mn_0.88_Zn_0.12_Br_4_. Figure S6: PL spectra of PEA_2_Mn_0.88_Zn_0.12_Br_4_ during heating process (a) and cooling process (b). The transformation of PEA_2_Mn_0.88_Zn_0.12_Br_4_ in CIE color coordinates during heating process (c) and cooling process (d). Figure S7: (a) FWHM, PL intensity and emission peaks of PEA_2_Mn_0.88_Zn_0.12_Br_4_ during 60 heating-cooling cycles. PL spectra of the PEA_2_Mn_0.88_Zn_0.12_Br_4_ at 140 °C (b) and 25 °C (c) at different heating and cooling cycles. Figure S8: (a) PL and PLE spectrum (b) XRD pattern of PEA_2_Pb_0.1_Mn_0.8_Zn_0.1_Br_4_. Figure S9: PL spectra of PEA_2_PbBr_4_ with different amounts of (a) MnAc_2_ and (b) MnBr_2_. (c) PL spectra of PEA_2_PbBr_4_ with different amounts of PEA_2_MnBr_4_. (d) PL spectra of PEA_2_MnBr_4_ with different amounts of PbBr_2_. Figure S10: (a) PL and (b) PLE spectra of PEA_2_MnBr_4_ with PbBr_2_ dissolved by different ligands. Table S3: Concentration ratio of Mn^2+^, Zn^2+^ and Pb^2+^ in the products by ICP-OES. Figure S11: Decay curves of PEA_2_Mn_0.88_Zn_0.12_Br_4_ doping Pb^2+^ in different levels. Figure S12: (a) XPS survey spectra of PEA_2_Mn_0.79_Zn_0.12_Pb_0.09_Br_4_ and PEA_2_Mn_0.88_Zn_0.12_Br_4_. High-resolutions XPS spectra of Zn2p (b) Mn2p (c) Pb4f (d) in PEA_2_Mn_0.79_Zn_0.12_Pb_0.09_Br_4_. Figure S13: Air stability (a) and thermal stability (b) of PEA_2_Mn_0.79_Zn_0.12_Pb_0.09_Br_4_. Figure S14: PL intensity and emission peaks of the multilayer composite fluorescent film during 10 heating-cooling cycles.
