# Supplementary material for: Metal-Ion-Doped Manganese Halide Hybrids with Tunable Emission for Advanced Anti-Counterfeiting
Source: Nanomaterials (Basel). 2023 Jun 20;13(12):1890. doi: 10.3390/nano13121890 (PMC10305547; doi:10.3390/nano13121890)
Supplement: Supplementary file 1 [file nanomaterials-13-01890-s001.zip › nanomaterials-2467519-supplementary.pdf]

Supplementary Materials

Metal-ion-doped Manganese Halide Hybrids with Tunable Emission for Advanced Anti-Counterfeiting

Experimental Section

Materials: Zinc acetate dihydrate ($ZnAc_2$, 99.9%), lead acetate ($PbAc_2$, 99.99%), manganese acetate ($MnAc_2$, 98%), β-phenylethylamine (PEA, 98%), tetra octyl ammonium bromide (TOAB, 98%), dodecylbenzene sulfonic acid (DBSA, 90%) and octanoic acid (OTAc, 99%) were purchased from Aladdin. Lead bromide ($PbBr_2$, 99.99%)was purchased from Macklin. Manganese (II) bromide ($MnBr_2$, 97%) was purchased from Strem. Bromotrimethylsilane (TMSBr, 97%) was purchased from J&K. Oleic acid (OA, 85%) was purchased from TCI. Xylene (95%) and hexane (97%) were purchased from Kermel.

Synthesis of PEA_2MnBr_4: 0.2 mmoL of $MnAc_2$, 80 μL PEA, 0.3 mL DBSA, 0.2 mL OTAc and 5 mL ODE were placed into a 10 mL three-necked flask. Then, the flask was subjected to vacuum-nitrogen three times and heated to 80 °C to dissolve. After that, the flask was heated to 140 °C, and 150μL TMSBr was injected into the flask. 5 s later, the solution was cooled by an ice-water bath. The crude solution was centrifugated at 5000 rpm for 3 min. After centrifugation, the precipitate was washed with xylene twice and dried under vacuum.

Synthesis of $PEA_2Mn_{0.88}Zn_{0.12}Br_4$: The synthetic procedure of $PEA_2Mn_{0.88}Zn_{0.12}Br_4$ was similar to that of pure PEA_2MnBr_4, except the addition of $ZnAc_2$. The new addition of $MnAc_2$ and $ZnAc_2$ are 0.0294 and 0.0066 g.

Synthesis of Pb^{2+}-doped $PEA_2Mn_{0.88}Zn_{0.12}Br_4$ by hot injection method: The synthetic procedure of Pb-doped $PEA_2Mn_{0.88}Zn_{0.12}Br_4$ was similar to that of $PEA_2Mn_{0.88}Zn_{0.12}Br_4$, except the addition of $PbAc_2$. The new addition of $MnAc_2$, $ZnAc_2$ and $PbAc_2$ are 0.0277, 0.0044 and 0.0065 g.

Synthesis of Pb^{2+}-doped $PEA_2Mn_{0.88}Zn_{0.12}Br_4$ by cation exchange method: 0.0037g $PbBr_2$ and 0.0109g TOAB were dissolved in 0.2 mL xylene to form lead bromide precursor. Then the lead bromide precursor was dropped into 3 mL of $PEA_2Mn_{0.88}Zn_{0.12}Br_4$ solution (0.124 g $PEA_2Mn_{0.88}Zn_{0.12}Br_4$ in 5 m L xylene). The solution was stirred for 10 min at room temperature. Then, the solution was subjected to centrifuge at 5000 rpm for 5 min. 5mL of Xylene was added to the precipitate, and then centrifuged at 5000 rpm for 5 min. After that, hexane was added to wash the precipitate.

Characterization: The PL and PLE spectra were conducted by a FLS920P spectrometer. The absolute PLQYs and time-resolved PL decays of the samples were measured by a fluorescence spectrometer (FLS920P, Edinburgh Instruments). The Fourier transform infrared (FTIR) spectrum was conducted on a Thermo-Nicole iS50 FTIR spectrometer with attenuated total reflection detector. The Inductively coupled plasma optical emission spectrometry (ICP-OES) measurements were measured by an ICP Optima 8300. X-ray photoelectron spectroscopy (XPS) was recorded on a Thermo Scientific K-Alpha spectrometer. The X-ray diffraction (XRD) measurements were measured on a Rigaku Smart Lab 9 kW. Field emission scanning electron microscope (FESEM) images were acquired using ZEISS Sigma 500.

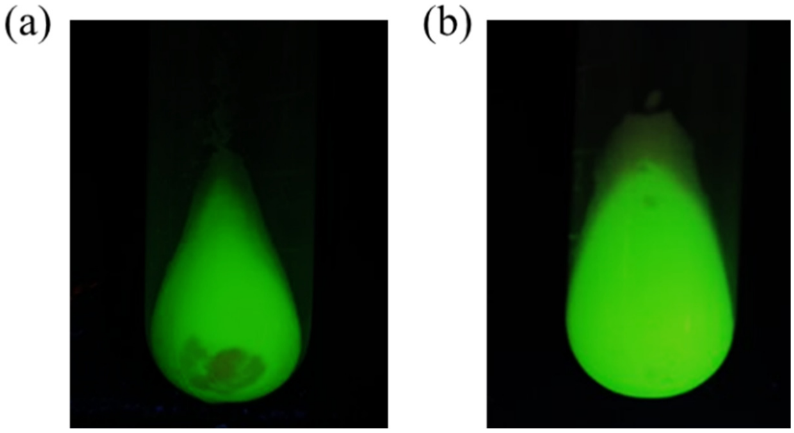

Figure S1 (a)The photograph of PEA_2MnBr_4 synthesized with DBSA. (b) The photograph of PEA_2MnBr_4 synthesized with DBSA and OTAc.

Table S1. Concentration ratio of Zn^{2+} in the products by ICP-OES

Sample (feed ratio % of Zn^{2+}) {[Zn] ⁄ ([Zn] + [Mn])}	ICP-OES (atomic ratio % of Zn^{2+}) {[Zn] / ([Zn] + [Mn])} ×100
5	4.6
15	12.3
25	23.6
35	32.9
50	45.7

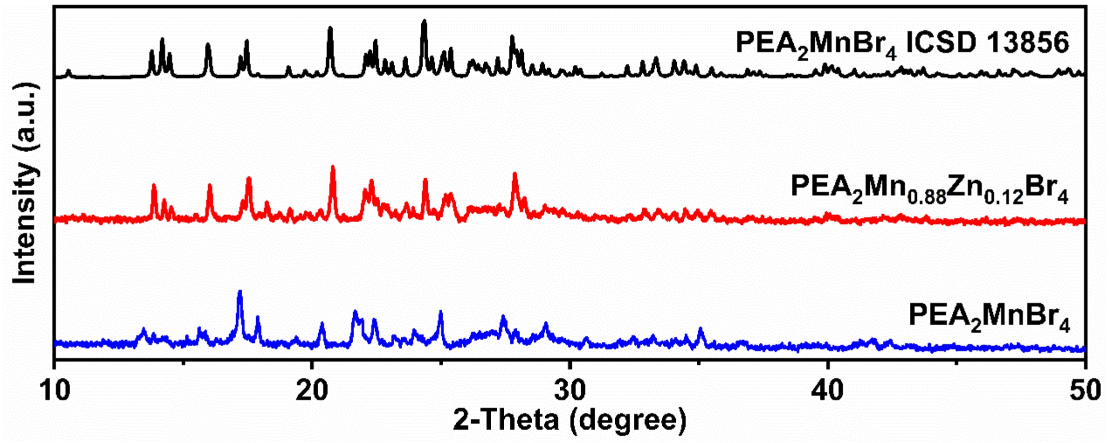

Figure S2 XRD patterns of PEA_2MnBr_4 and $PEA_2Mn_{0.88}Zn_{0.12}Br_4$

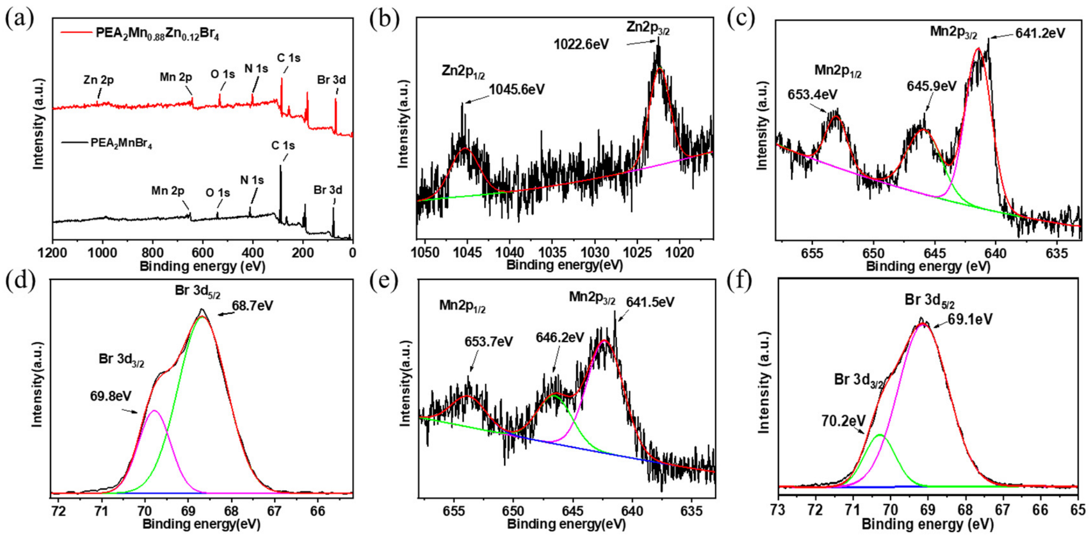

Figure S3 (a) XPS survey spectra of PEA_2MnBr_4 and $PEA_2Mn_{0.88}Zn_{0.12}Br_4$. High-resolutions XPS spectra of Zn2p (b) Mn2p (c) and Br3d (d) in $PEA_2Mn_{0.88}Zn_{0.12}Br_4$. HR-XPS spectra of Mn2p (e) and Br3d (f) in PEA_2MnBr_4.

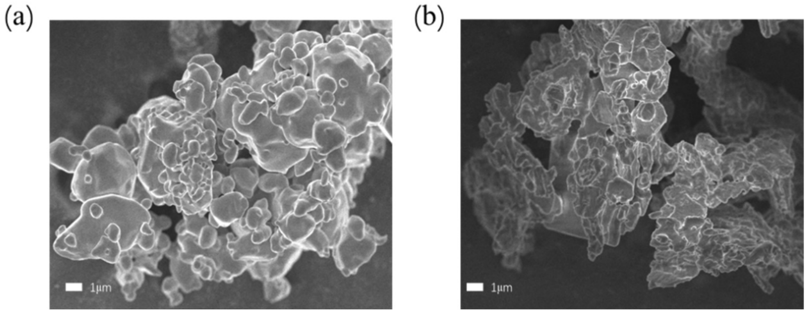

Figure S4 SEM images of PEA_2MnBr_4 (a) and $PEA_2Mn_{0.88}Zn_{0.12}Br_4$ (b).

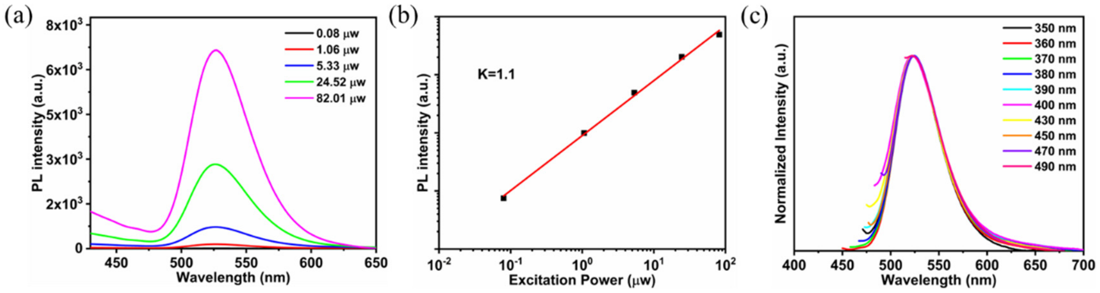

Figure S5 (a) Excitation power-dependent PL spectra for an excitation wavelength of 365 nm. (b) Integrated PL intensities for the emission centered at 528 nm of the $PEA_2Mn_{0.88}Zn_{0.12}Br_4$ in relation to the excitation power (0.08-82 μW). (c) Excitation wavelength-dependent PL spectra of $PEA_2Mn_{0.88}Zn_{0.12}Br_4$.

Table S2. Excitation and crystal field parameters for PEA_2MnBr_4 and $PEA_2Mn_{0.88}Zn_{0.12}Br_4$

Sample	PEA_2MnBr_4	$PEA_2Mn_{0.88}Zn_{0.12}Br_4$
$^4E(D)$ (nm)	375.8	376.1
$^4A_1$, $^4E(G)$ (nm)	437	439
$^4T_1(G)$ (nm)	468	468.2
B (cm^{-1})	532.4	544.2
C (cm^{-1})	3511.9	3467.4
E_2 (cm^{-1})	21367.5	21358.4
Dq (cm^{-1})	327.5	343.3

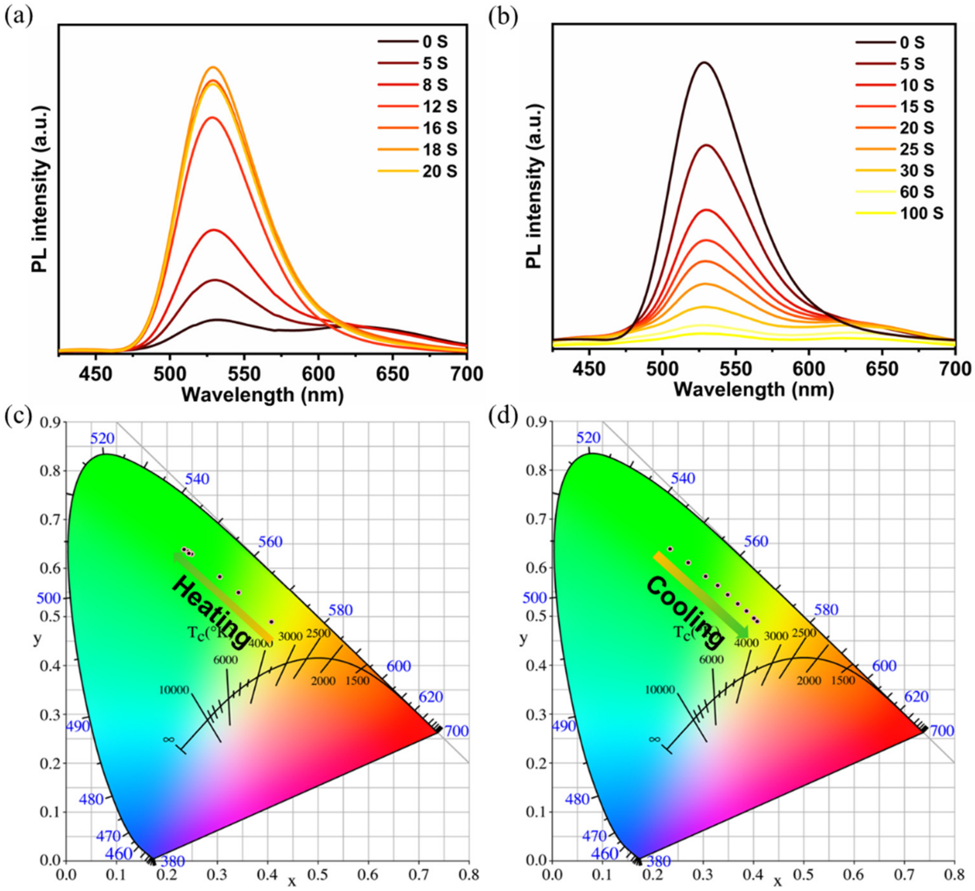

Figure S6 PL spectra of $PEA_2Mn_{0.88}Zn_{0.12}Br_4$ during heating process (a) and cooling process (b). The transformation of $PEA_2Mn_{0.88}Zn_{0.12}Br_4$ in CIE color coordinates during heating process (c) and cooling process (d).

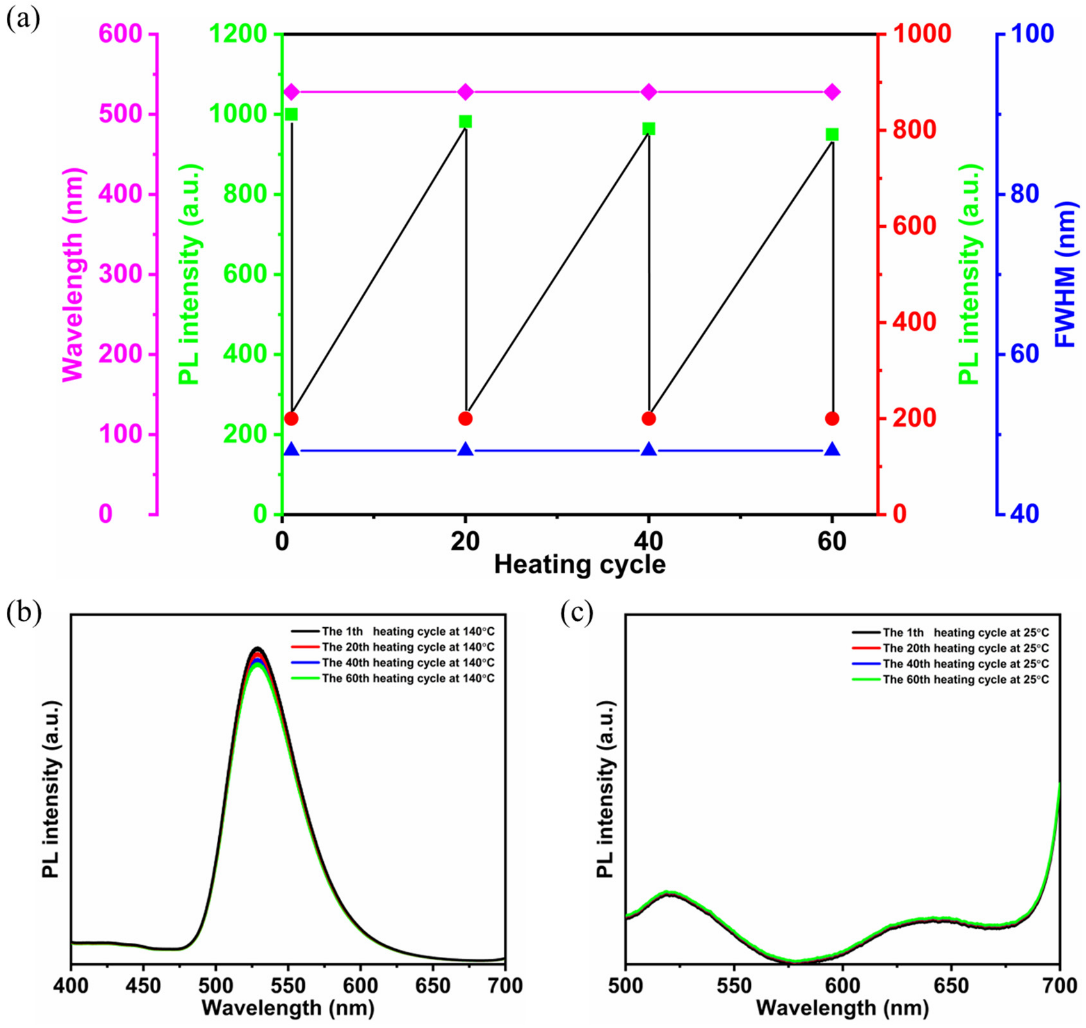

Figure S7 (a) FWHM, PL intensity and emission peaks of $PEA_2Mn_{0.88}Zn_{0.12}Br_4$ during 60 heating - cooling cycles. PL spectra of the $PEA_2Mn_{0.88}Zn_{0.12}Br_4$ at 140 ℃ (b) and 25 ℃ (c) at different heating and cooling cycles.

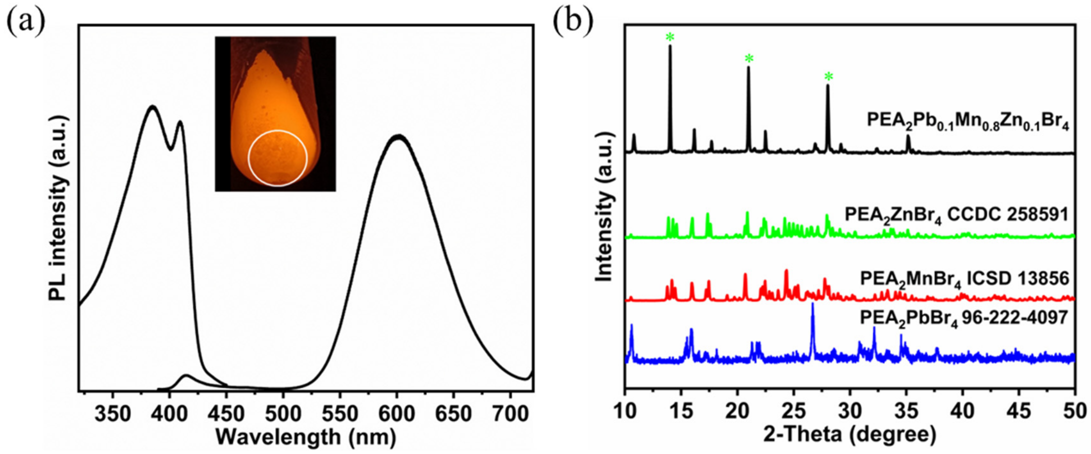

Figure S8 (a) PL and PLE spectrum (b) XRD pattern of $PEA_2Pb_{0.1}Mn_{0.8}Zn_{0.1}Br_4$

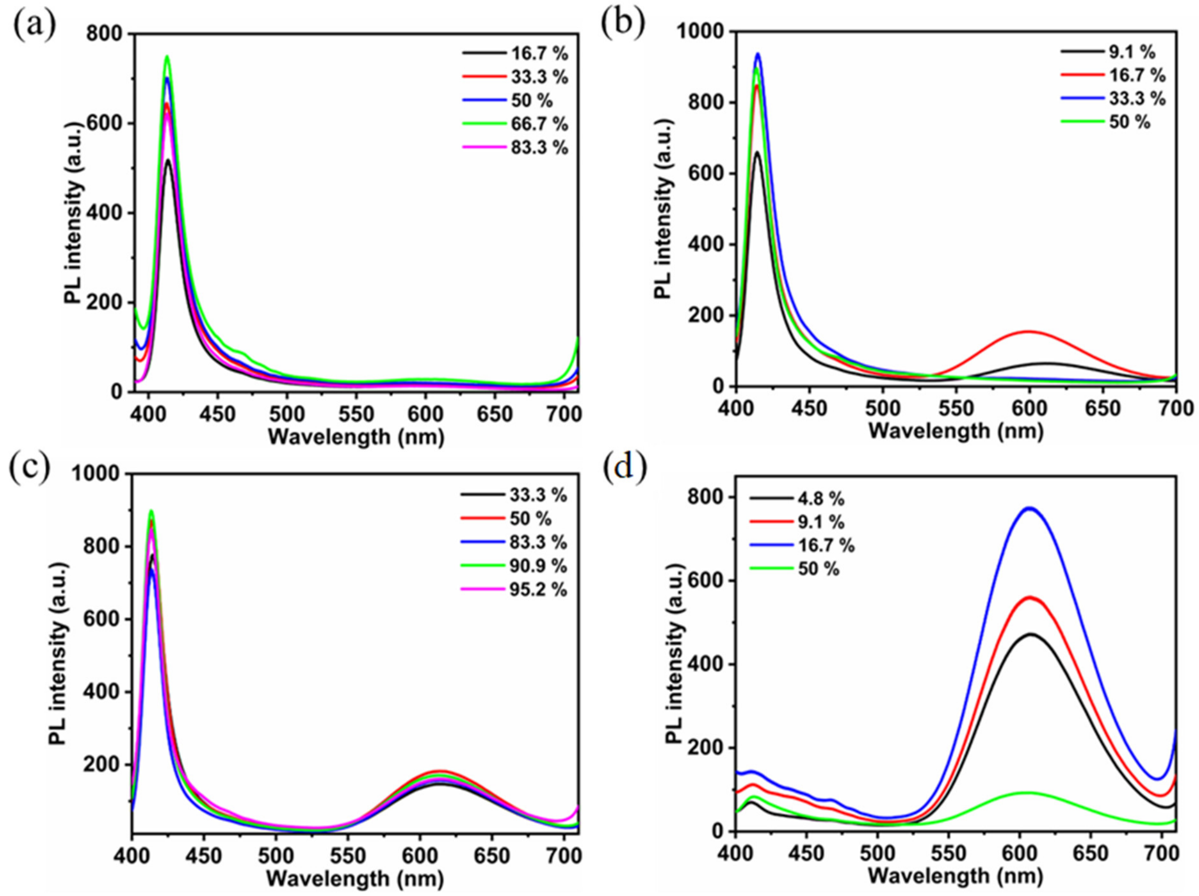

Figure S9 PL spectra of PEA_2PbBr_4 with different amounts of (a) $MnAc_2$ and (b) $MnBr_2$. (c) PL spectra of PEA_2PbBr_4 with different amounts of PEA_2MnBr_4. (d) PL spectra of PEA_2MnBr_4 with different amounts of $PbBr_2$.

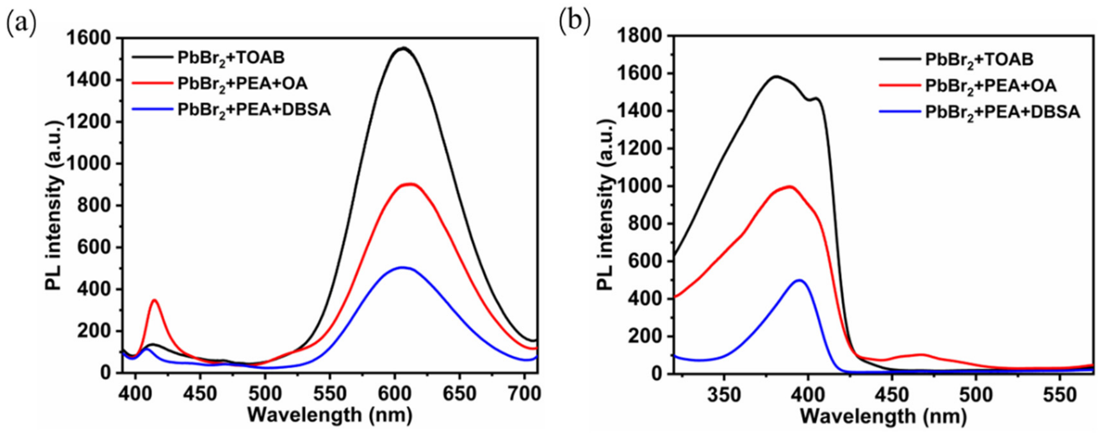

Figure S10 (a) PL and (b) PLE spectra of PEA_2MnBr_4 with $PbBr_2$ dissolved by different ligands.

TableS3. Concentration ratio of Mn^{2+}, Zn^{2+} and Pb^{2+} in the products by ICP-OES

Element / Sample (feed ratio % of Pb^{2+})	atomic ratio % of Mn^{2+}	atomic ratio % of Zn^{2+}	atomic ratio % of Pb^{2+}
6.3	81.2	11.9	6.9
9.1	78.9	11.7	9.4
16.7	72.6	10.6	16.8
88.9	10.8	1.6	87.6
95.2	5.8	0.5	93.7

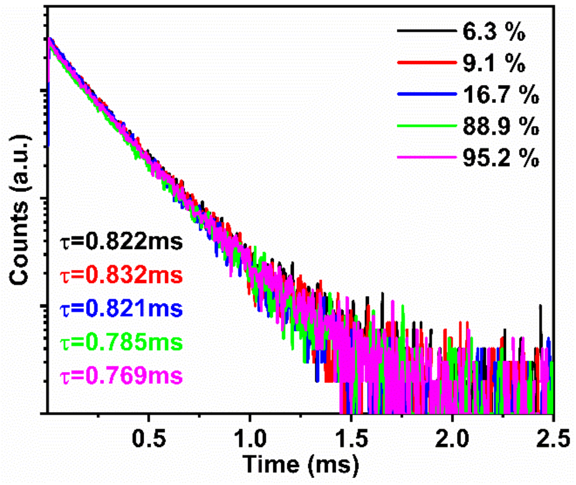

Figure S11 Decay curves of $PEA_2Mn_{0.88}Zn_{0.12}Br_4$ doping Pb^{2+} in different levels.

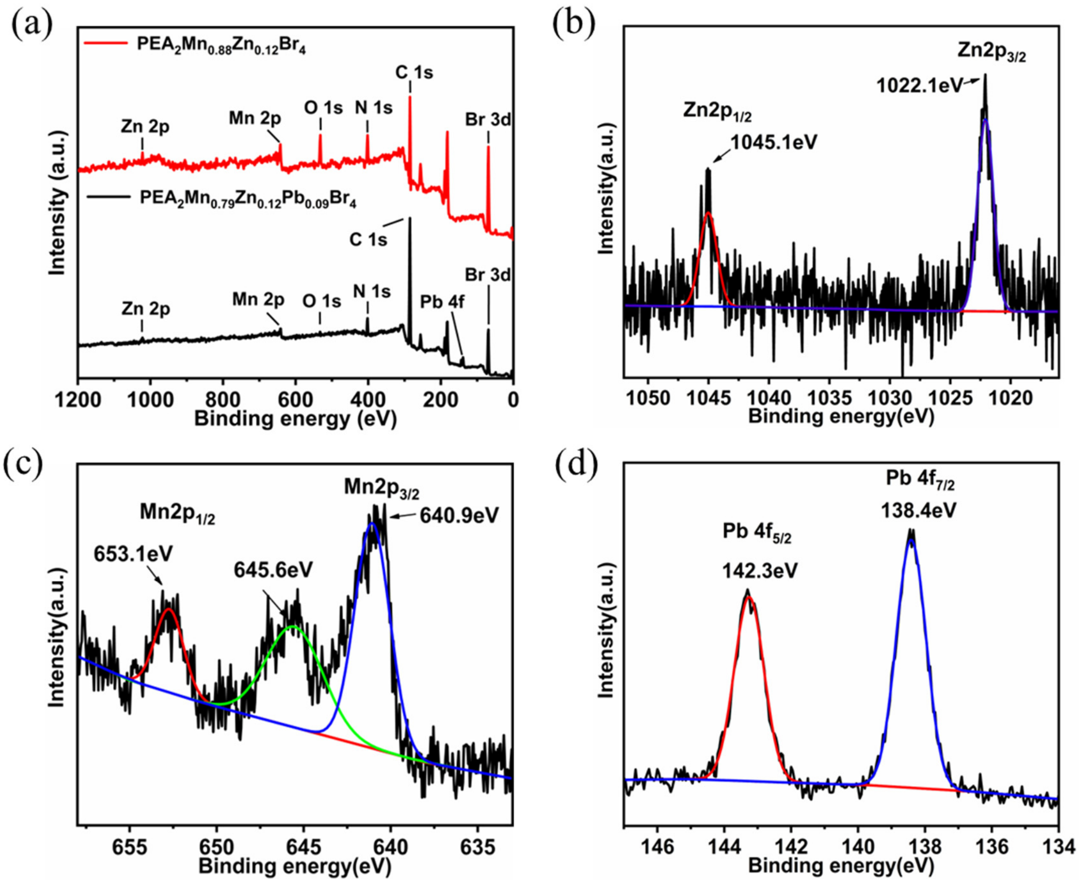

Figure S12 (a) XPS survey spectra of $PEA_2Mn_{0.79}Zn_{0.12}Pb_{0.09}Br_4$ and $PEA_2Mn_{0.88}Zn_{0.12}Br_4$. High-resolutions XPS spectra of Zn2p (b) Mn2p (c) Pb4f (d) in $PEA_2Mn_{0.79}Zn_{0.12}Pb_{0.09}Br_4$.

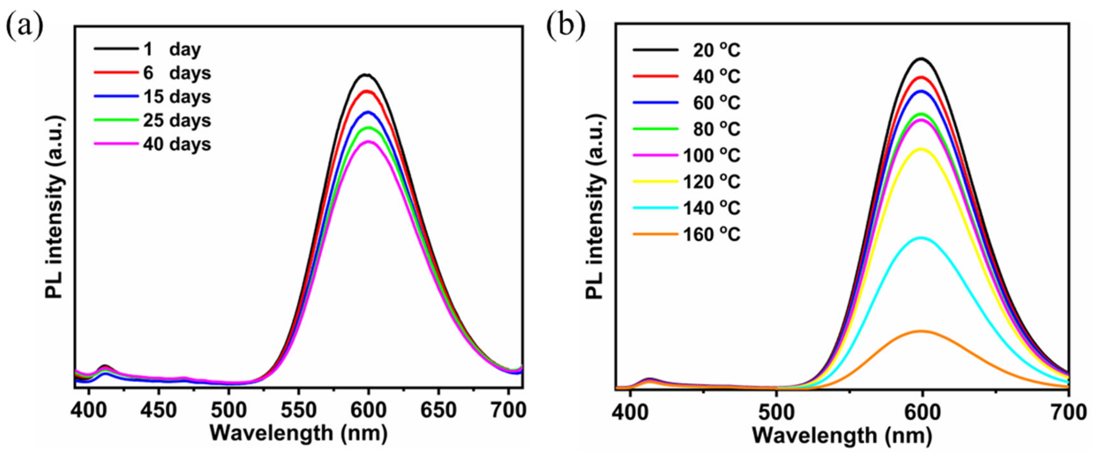

Figure S13 Air stability (a) and thermal stability (b) of $PEA_2Mn_{0.79}Zn_{0.12}Pb_{0.09}Br_4$.

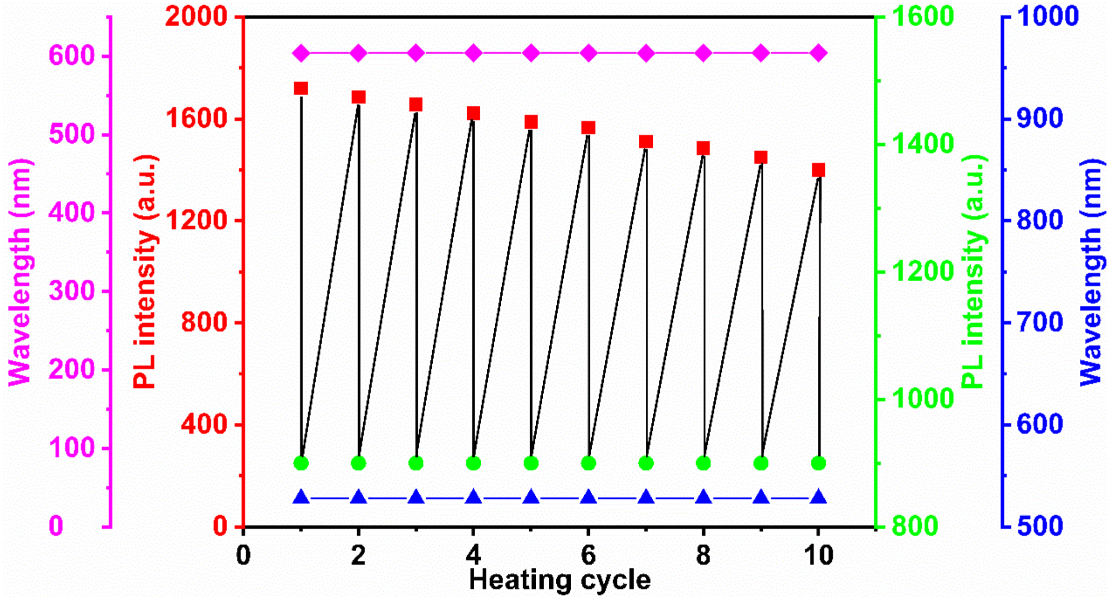

Figure S14 PL intensity and emission peaks of the multilayer composite fluorescent film during 10 heating - cooling cycles.
